# Amino Acid Profiles to Differentiate White Wines from Three Autochtonous Galician Varieties

**DOI:** 10.3390/foods9020114

**Published:** 2020-01-21

**Authors:** José Manuel Mirás-Avalos, Yolanda Bouzas-Cid, Emiliano Trigo-Córdoba, Ignacio Orriols, Elena Falqué

**Affiliations:** 1 Estación de Viticultura e Enoloxía de Galicia (EVEGA-AGACAL), Ponte San Clodio s/n, 32428 Leiro–Ourense, Spain; yolanda.bouzas.cid@xunta.gal (Y.B.-C.); emilianotrigo@hotmail.com (E.T.-C.); ignacio.orriols.fernandez@xunta.gal (I.O.); 2 Servizo de Prevención e Análise de Riscos, Dirección Xeral de Innovación e Industrias Agrarias e Forestais, Rúa Roma 25-27, 15703 Santiago de Compostela, Spain; 3 Depto. Química Analítica, Facultad de Ciencias, Universidade de Vigo, As Lagoas s/n, 32004 Ourense, Spain; efalque@uvigo.es; 4 Clúster de Investigación y Transferencia Agroalimentaria del Campus Auga (CITACA), As Lagoas s/n, 32004 Ourense, Spain

**Keywords:** nitrogen fraction, vine water status, *Vitis vinifera* (L.), wine classification

## Abstract

Amino acids play a relevant role in wine quality and can allow for classifying wines according to the variety. In this work, the amino acid contents of Albariño, Godello, and Treixadura wines, three autochthonous varieties from Galicia (NW Spain), were determined. During three consecutive vintages, these varieties were grown on the same vineyard and were harvested at optimum maturity, and the wines were elaborated following the same enological protocol. The identification and quantification of the primary amino acids were carried out by high-performance liquid chromatography with photodiode array detection, after a derivatization. Amino acid contents in these white varieties were within the range of values reported for other European wines, but Treixadura wines showed the highest concentrations, while wines from the Albariño variety showed the lowest contents. Apart from proline, whose concentrations were caused by yeast release, the most abundant amino acids were aspartic acid, glutamic acid, lysine, arginine, asparagine, alanine, and histidine. Principal component analysis separated wines by variety according to their amino acid contents.

## 1. Introduction

The amino acids present in grapes are consumed by yeasts during alcoholic fermentation and might yield some higher alcohols, aldehydes, esters, and other volatile compounds [1], influencing the final wine aroma [2]. Amino acids represent up to 40% of the total nitrogen in wines [3], and yeasts excrete other amino acids at the end of fermentation [4]; they are released by yeast autolysis or produced by enzymatic degradation of grape proteins. Furthermore, amino acid nature and concentrations in grapes depend on a wide range of factors, such as fertilization, climatic conditions, and grape variety [1], but the amino acid profiles were successfully employed by several researchers for differentiating and classifying grapes or wines from different varieties, management conditions and growing regions [5,6]. For instance, Soufleros et al. [7] were able to classify French wines from several terroirs (Bordeaux, Bourgogne, Alsace, Champagne) according to their origin, type, and aging through the analysis of 21 amino acids, biogenic amines, and volatile substances. In addition, Arrieta and Prats-Moya [8] reported that the growing region altered the amino acid concentrations in Monastrell wines. Moreover, Martínez-Pinilla et al. [9] differentiated red wines from Tempranillo, Monastel and Maturana Tinta de Navarrete according to grape variety, malolactic fermentation and vintage; concluding that wines from Tempranillo were less affected by vintage than those from the other varieties.

In Galicia (NW Spain), white wines are predominantly produced with autochthonous varieties. Albariño, Godello, and Treixadura are the most relevant varieties and have different and well-defined aroma and sensory properties. Hence, Albariño presents a high aromatic profile characterized by floral and fruity odors [10]; Treixadura wines are balanced and with a high aromatic potential [11]; Godello wines are structured but less aromatic than those from the other two varieties [10]. Recent studies determined the effect of irrigation on the amino acid composition of the musts from these varieties [12,13,14]; however, the amino acid profile of wines made from these varieties has never been characterized in order to differentiate the product.

Research efforts proved that the effect of the grape variety is one of the main determinants of the amino acid composition of wines [5,9]; however, no research described the amino acid profiles of several varieties grown on the same region and their wines made following the same procedure. Since amino acids have a relevant role in the progress of alcoholic fermentations and in the formation of aroma compounds, the current research aimed at: (1) Identifying amino acid profiles of wines from the three main grapevine white varieties grown in Galicia (NW Spain), and (2) characterizing and differentiating the wines from each variety according to their amino acid profiles.

## 2. Materials and Methods

### 2.1. Description of the Experimental Vineyard

The experiment was conducted over three consecutive years (2012-2014) in a 0.2 ha vineyard within the farm of the Estación de Viticultura e Enoloxía de Galicia in Leiro (42°21.6″ N, 8°7.02″ W, elevation 115 m), Ourense, Spain, within the Ribeiro Designation of Origin (DO).

Climate at this site is warm-temperate, moderately dry and with cold nights [15], with an average annual rainfall of 900 mm of which 70% falls during the dormant period. Over the growing season (April to harvest), rainfall was 313, 163, and 185 mm for 2012, 2013, and 2014, respectively. Moreover, the mean temperature over the growing season increased from year to year. During the maturation period (August and September), the highest temperatures were recorded in 2013. In addition, no rainfall events occurred in August 2013, while more than 20 mm rainfall were registered in 2012 and 2014. Until harvest, September was dry in all the studied years, and rainfall ranged from 0.2 mm in 2012 to 12.4 mm in 2014 (Table 1).

Soil at this site is sandy textured (64% sand, 16.4% silt, and 19.6% clay), slightly acidic (pH = 6.3), and of medium fertility (2.7% organic matter). The soil is rather shallow (≈1.2 m) and its available water capacity is 100 mm m^−1^, approximately.

The vineyard was planted with three white grapevine (*Vitis vinifera* L.) varieties autochthonous from NW Spain: Albariño, Godello, and Treixadura. All of them were grafted in 1998 on 196-17C rootstock. Vines were vertically trellised on a single cordon system (10–12 buds per vine). Rows were east–west oriented; spacings were 1.25 and 2.4 m between vines and rows, respectively (3333 vines ha^−1^). No fertilization was applied to the vineyard during the study period.

### 2.2. Field Determinations

Grapevine water status was assessed every two weeks through the measurement of stem water potential (Ψ_stem_) at midday (12 h–13 h) using a pressure chamber (Pump-Up, PMS, Albany, OR, USA). These determinations were carried out on an adult leaf from nine vines per variety. Leaves were covered with aluminum foil 1 h prior to the readings [16]. The water stress integral that expresses the severity by duration of the stress above a minimum value was calculated using the Ψ_stem_ data from each treatment and year, as defined by Myers [17].

Harvest was performed in mid-September for all varieties and years. Respectively for 2012, 2013 and 2014, harvest dates were 13, 19, and 11 September for Albariño; 11, 17, and 9 September for Godello; and 21, 23, and 15 September for Treixadura. In those dates, grape yield was determined by counting and weighting all clusters from 24 vines per variety (three replications of eight vines each and located in different areas of the vineyard). Pruning weight at winter was determined on 18 vines per variety (three replications of six vines each and located in different areas of the vineyard).

### 2.3. Sampling and Winemaking

Grapes from the different varieties were manually harvested at their optimal maturity. Winemaking was performed separately on lots of 40 kg, approximately, per replicate (hence, 3 lots per variety and year) as detailed in Trigo-Córdoba et al. [18].

In summary, grapes from each replicate were destemmed, crushed and pressed in a pneumatic press, and then, pectolytic enzyme was added to favor settling (4 g per 100 kg of grapes). A replicated sample (250 mL) was collected for determining the basic parameters of musts (total soluble solids, pH, total acidity and the concentrations of malic and tartaric acids) following the official methods [19].

SO_2_ (50 mg L^−1^) was added and, after 24 h, musts were racked and moved to 35-L stainless steel tanks for alcoholic fermentation with a commercial yeast (Excellence FW, Lamothe-Abiet, Bordeaux, France), added at 20 g h L^−1^. Density and temperature of fermentations were monitored daily. Once alcoholic fermentation finished, wines were racked and SO_2_ was added to 35 mg L^−1^ free sulfur dioxide. A natural clarification at 4 °C was carried out for one month. Finally, wines were filtered, bottled and stored for five months at 10 °C until analysis. The basic parameters of wines (alcohol content, pH, total acidity, and the concentrations of malic and tartaric acids) were determined by Fourier transform infrared spectrometry (FTIR) using a WineScan FT120 analyzer (FOSS Electric, Barcelona, Spain) calibrated according to the official methods [19].

### 2.4. Determination of Free Amino Acids in the Wines

The free amino acid contents were determined in triplicate five months after wine bottling through high-performance liquid-chromatography (HPLC) following a method based on a previous derivatization reaction [6,20] with slight modifications [12]. Briefly, amino acids were determined after reaction of 1.75 mL of borate buffer 1 M (pH = 9), 0.75 mL of methanol, 1 mL of sample wine without any pre-treatment, 20 µL of internal standard (L-2-aminoadipic acid, 1 g L^−1^), and 30 µL of the reagent for derivatization, diethylethoxymethylenemalonate (DEEM) (Acros Organics, New Jersey, USA) in a screw-cap tube over 30 min in an ultrasound bath. Then, the sample was heated at 70 °C for 2 h to degrade the excess of DEEM and reagent by-products.

The HPLC analysis was conducted on an 1100 Series equipment (Agilent Technologies, Palo Alto, CA, USA), by using a Zorbax Eclipse AAA column (C18), particle size 5 µm (150 mm × 4.6 mm; Agilent) with a pre-column (Zorbax Eclipse AAA, 12.5 mm × 4.6 mm; Agilent). This column was thermostated at 22 °C. The injected volume was 50 µL and a photodiode array detector (DAD) was used at 280 nm for amino acids detection.

The mobile phase A was 25 mM acetate buffer (pH 5.8) with 0.4 g of sodium azide; the mobile phase B was acetonitrile and methanol (80:20, v/v) (super-gradient HPLC grade acetonitrile and methanol from Scharlau, Sentmenat, Spain). Elution conditions were as follows: 0.8 mL min^−1^ flow rate, 10% B during 20 min, then elution with linear gradients from 10 to 17% B in 10 min, from 17 to 19% in 0.01 min, maintained during 0.99 min, from 19% to 19.5% B in 0.01 min, from 19.5% to 23% in 8.5 min, from 23% to 29.4% B in 20.6 min, from 29.4% to 72% B in 8 min, from 72% to 82% B in 5 min, from 82% to 100% B in 4 min, maintained during 3 min, followed by washing and reconditioning the column.

These chromatographic conditions allowed the separation, identification and quantification of ammonium ion (ammonium chloride was from Merck, Darmstadt, Germany) and 22 amino acids (Acros Organics, New Jersey, NJ, USA), which, by alphabetical order, were the following: Alanine (Ala), Asparagine (Asp), Aspartic acid (Aspacid), Arginine (Arg), Cysteine (Cys), γ-aminobutyric acid (GABA), Glutamic acid (Gluacid), Glutamine (Glu), Glycine (Gly), Histidine (His), Isoleucine (Ile), Leucine (Leu), Lysine (Lys), Methionine (Met), Ornithine (Orn), Phenylalanine (Phe), Proline (Pro), Serine (Ser), Threonine (Thr), Tryptophan (Try), Tyrosine (Tyr), and Valine (Val).

These compounds were identified according to the retention times and to the UV-vis spectral characteristics of the derivatives of the corresponding standards. The quantification was performed using the internal standard method. The detection (LOD) and quantification (LOQ) limits of the different compounds were calculated as three and ten times, respectively, the standard deviation provided by the signal noise ratio in the lowest concentrations [12,13]. The LOD values were lower than 0.1 mg L^−1^.

### 2.5. Statistical Evaluation

Grapevine variety, year and their interaction were used as factors for analyzing data by two-way analysis of variance (ANOVA). When needed, mean separation was carried out using the Tukey’s Honest Significance Difference (HSD) test. Differences were considered significant when *p*-values were lower than 0.05. Principal component analysis (PCA) was applied to the amino acid concentrations to separate the wines according to variety and year. Statistical procedures were performed using R software v3.6.1 [21].

## 3. Results

### 3.1. Grapevine Water Status, Vegetative Growth, and Yield

**Figure 1:**
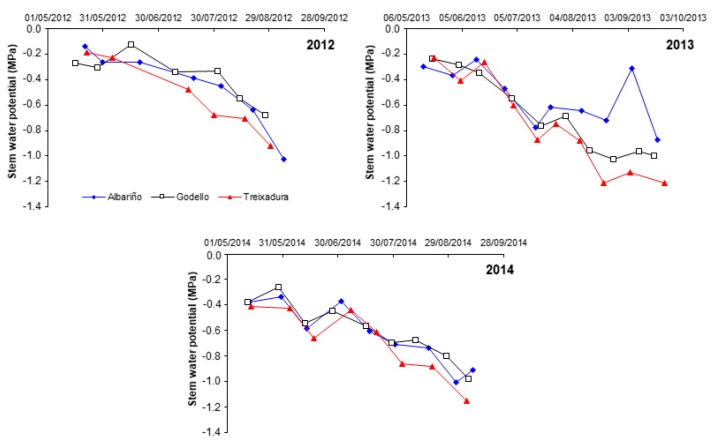
Dynamics of the midday stem water potential over the growing season for Albariño, Godello and Treixadura vines in the years 2012, 2013, and 2014. Each data is the average of 9 measurements.

Figure 1 shows the evolution of Ψ_stem_ over the growing season for the three varieties considered in the current study. From August onwards, Treixadura showed more negative values (−1.2 MPa) than Albariño and Godello (−1 MPa). The most negative values of Ψ_stem_ were observed on dates close to harvest, although the vines only suffered from a slight to moderate water stress over the three studied years. For the three varieties, Ψ_stem_ values were less negative in 2012 when compared to those measured in 2013 and 2014.

**Figure 2:**
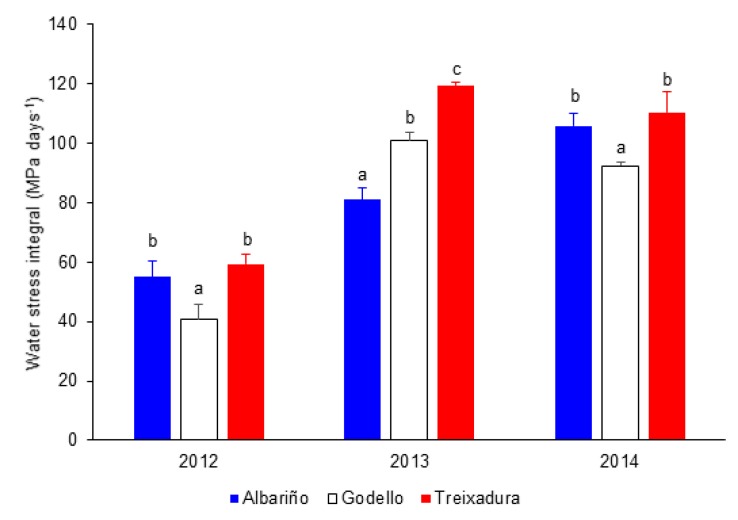
Water stress integral values for the Albariño, Godello, and Treixadura vines in the years 2012, 2013, and 2014. Letters over the bars indicate significant differences among varieties for a given year. Error bars represent standard errors.

When considering the values accumulated for the entire growing season (Figure 2), Treixadura presented the highest water stress integral in the three years studied, although in 2102 and 2014 the difference was not significant with Albariño. In 2013, Albariño suffered from less water stress intensity than Godello and Treixadura, which was the variety most affected by this abiotic stress (Figure 2).

Regarding the productive response of grapevines, the number of clusters per vine was lower for Treixadura than for Albariño and Godello (Table 2). In contrast, Godello showed a significant (*p* < 0.05) higher yield (5.4 kg vine^−1^) than the other two varieties, which had similar yields. Cluster weight was different among the three varieties; Albariño had the lightest clusters while Treixadura presented the heaviest ones. Pruning weight was significantly lower for Treixadura than for the rest of the studied varieties. The effect of the year was significant for all the yield components and pruning weight. However, the interaction between year and variety was significant only for yield (Table 2).

### 3.2. Basic Parameters of Musts and Wines

The general parameters of the musts differed among varieties, except for the total soluble solids content (Table 3). The musts from Albariño had the highest acidity, whereas those from Treixadura had the lowest one. The concentration of malic acid was significantly lower in the musts from Godello and that of tartaric acid was lower in musts from Treixadura. Year exerted a significant effect on the malic acid concentration. In addition, the interaction between variety and year was significant for the tartaric acid concentration in the musts (Table 3).

Except for alcohol content, the general parameters of the wines differed significantly among varieties (Table 4). Wines from Albariño had the highest acidity, whereas those from Treixadura had the lowest one, and the contrary occurred for pH. The concentration of malic acid was lower in the wines from Godello and that of tartaric acid was higher in wines from Albariño. Year exerted a significant effect on wine pH and malic acid concentration. No significant interactions between year and variety were detected for any of the parameters considered (Table 4).

### 3.3. Amino Acids in Wines

The average concentration of free amino acids, without proline because this amino acid is excreted by yeasts, in the wines from the three studied varieties showed values from 59.5 mg L^−1^ to 159.8 mg L^−1^ for the three studied years (Table 5). Although the same compounds were detected in wines from the three varieties, the variety exerted a significant influence on the amino acid concentrations (Table 5).

In general, Albariño wines had the lowest and Treixadura the highest concentrations of amino acids, with Godello wines having an intermediate behavior. However, Cys was not significantly affected by the variety. In contrast, 14 compounds (Aspacid, Asp, Glu, His, Gly, Thr, Arg, GABA, Pro, Tyr, Val, Ile, Try, and Orn) were significantly higher in Treixadura wines than in those from Albariño and Godello (Table 5). Five compounds (Ser, Ala, Leu, Phe, and Lys) appeared in the following rank order: Treixadura > Godello > Albariño (Table 5). Gluacid concentration was similar in Godello and Treixadura wines, both higher than Albariño (Table 5). Seven amino acids were significantly affected by year (Table 5). These amino acids included Gluacid, Aspacid, Ser, Arg, GABA, Tyr, and Try. In general, amino acid concentrations in wines were lower in 2013 than in the other studied years (Supplementary Tables S1–S3). The interannual variability in the concentrations of amino acids in wines was lower in Albariño (Supplementary Table S1) than in Godello (Supplementary Table S2) and Treixadura (Supplementary Table S3). The interaction between year and variety exerted a significant influence on Tyr concentration (Table 5).

When considering the intervals between the minimum and maximum concentrations for each amino acid in wines from the three varieties studied (Table 6), it became clear that Treixadura presented the highest concentrations (both maximal and minimal) for most of the individual compounds determined, while wines from Albariño tended to have the lowest concentrations, being Godello wines those with intermediate concentrations. Nevertheless, some amino acids showed ranges of concentrations overlapped among the three varieties; these compounds included Gluacid, Asp, Glu, His, Gly, Thr, Arg, Tyr, Met, Cys, Try, and Orn (Table 6). Interestingly, Cys was only detected in Treixadura wines, although not all years (Supplementary Table S3). Moreover, the maximum concentrations of 10 amino acids (Aspacid, Ser, Ala, GABA, Pro, Val, Ile, Leu, Phe, and Lys) in Albariño wines were lower than the minimum concentrations in Treixadura wines (Table 6).

**Figure 3:**
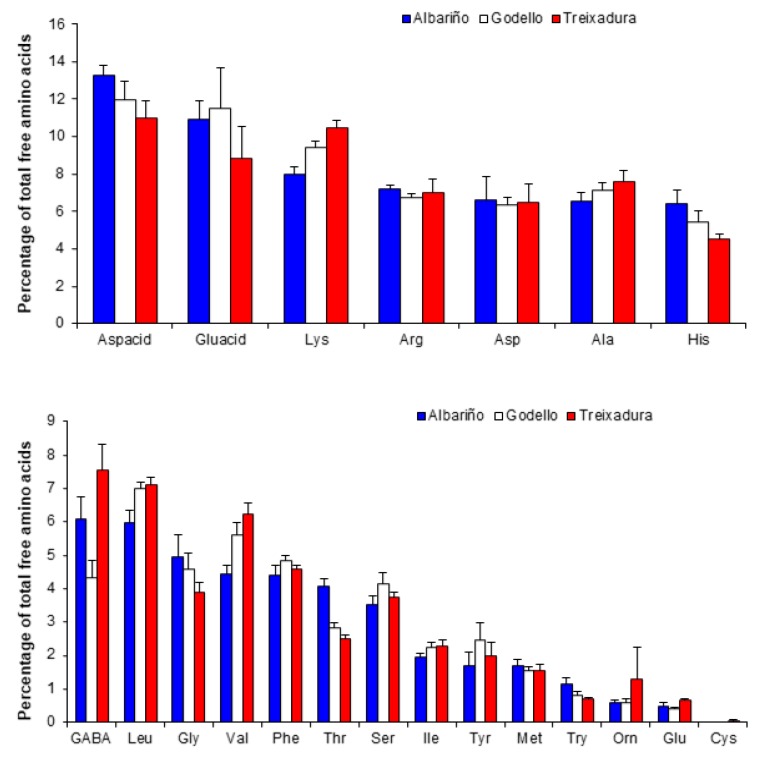
Average percentage (three years) of each amino acid over the total free amino acids in Albariño, Godello, and Treixadura wines. Bars indicate standard errors. Abbreviations: Aspacid (aspartic acid), Gluacid (glutamic acid), Lys (lysine), Arg (arginine), Asp (asparagine), Ala (alanine), His (histidine), GABA (aminobutyric acid), Leu (leucine), Gly (glycine), Val (valine), Phe (phenylalanine), Thr (threonine), Ser (serine), Ile (isoleucine), Tyr (tyrosine), Met (methionine), Try (tryptophan), Orn (ornithine), Glu (glutamine), Cys (cysteine).

When Pro is not considered, Aspacid was the most abundant amino acid in wines from the three studied varieties, accounting for 11%–13% of the total free amino acids (Figure 3). In the three varieties, Gluacid was abundant, although in Treixadura its percentage did not reach 10%. In contrast, Lys was more abundant in Treixadura than in Albariño wines. Arg, Asp, and Ala percentages were very similar among varieties (Figure 3). Finally, His percentage was higher in Albariño than in Treixadura wines.

The percentage of GABA and Leu is around 7% in Treixadura and 6% in Albariño and Godello wines (Figure 3). In Albariño samples, the percentages of Gly, Thr, and Try were higher, whereas those of Val, Orn, and Glu were higher in Treixadura wines. Godello wines showed slightly higher percentages of Phe, Ser, and Tyr. The rest of the amino acids detected in the wines had similar percentages among the varieties (Figure 3).

**Figure 4:**
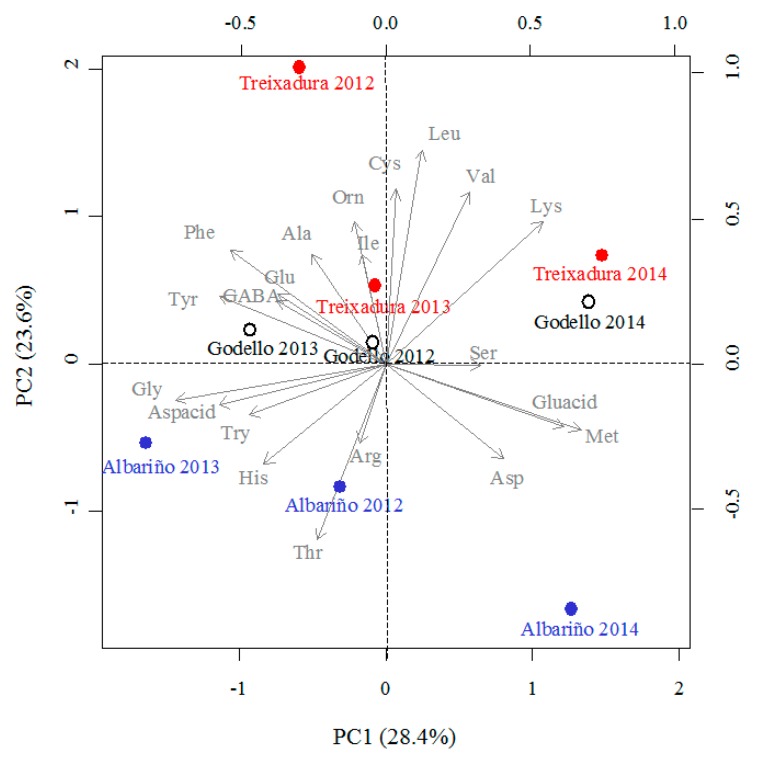
Principal component analysis (PCA) of Albariño, Godello and Treixadura wines from 2012, 2013, and 2014. Bi-plot for the first two components (PC) for free amino acids except proline.

The percentages of free amino acids, except Pro contents, were submitted to a PCA, and Figure 4 revealed a clear separation of the wines made with each variety and year. The first two principal components accounted for 52% of the total variance in the data set: PC1 explained 28.4% of this variance and PC2 explained 23.6%. Independently of the variety, PC1 separated wines from 2014, located on the positive side, from those produced in 2012 and 2013, located on the negative side (Figure 4). Moreover, PC2 separated wines made with Albariño, located on the negative side of this PC, from those made with Godello and Treixadura, which appeared on the positive side of this PC (Figure 4).

The amino acids that contributed positively to the construction of PC1 were Ser, Gluacid, Met and Lys. In contrast, Gly, Aspacid, Try, Tyr, and GABA contributed negatively to construct PC1. Furthermore, Orn, Cys, Ile, Leu, Val, and Ala contributed positively to construct PC2, whereas Arg and Thr contributed negatively (Figure 4).

## 4. Discussion

The three grapevine varieties studied were grown on the same vineyard and using the same agricultural practices; however, their performance was different. For instance, Albariño and Godello showed less negative midday stem water potential values over the growing season when compared with Treixadura, which reached levels of moderate water stress [22]. This different water status among varieties made that Treixadura had less clusters per plant (but they were heavier) and a lower pruning weight than the other two varieties, whereas Godello reached the highest yields per plant. This response is typical from cultivars suffering from a moderate degree of water stress [23]. However, this did not impede that grapes from all varieties reached an adequate level of maturation, as proven by the similar total soluble solids values among varieties. However, Albariño musts were more acidic than those from Godello and Treixadura, as previously reported [24]. These differences were maintained in the wines.

Depending on the year and variety, the total primary amino acid concentration for Albariño, Godello and Treixadura wines ranged from 37.5 to 269.8 mg L^−1^ when Pro is not considered (Supplementary Tables S1–S3). However, it can reach up to 3266.8 mg L^−1^, when Pro is taken into account. These concentrations were lower in 2013 than in the other studied years, likely due to a most intense water stress [2,23] caused by the environmental conditions of that given year. This reduction in amino acid concentrations due to water stress has also been observed for other varieties [9].

On average for the three considered harvests, the concentration of amino acids was 59.5 mg L^−1^ for Albariño wine, 89.0 mg L^−1^ for Godello wine and 159.8 mg L^−1^ for Treixadura wine. This last variety presented the triple content of free amino acids than Albariño, but these average concentrations observed in Albariño wines were slightly lower than the values reported for white wines from other regions [5,7,25,26] and much lower than those listed for wines elaborated from red varieties [8,9,27,28]. The most abundant amino acids in these Galician wines were aspartic and glutamic acids, Lys, Arg, Asp, Ala, and His, representing about the 58% of the total primary amino acid content. Some of them appeared in significant concentrations in wines from other varieties [5,9,27]. For instance, a previous study on several white varieties [5] showed that the most abundant amino acids in wines from Roditis were Arg and Lys; Arg, Lys, and Glu for Muscat d’Alexandrie; Ala and Thr for Muscat white; Arg, Ala, Glu and GABA for Chardonnay. The three Galician varieties reported here showed a different amino acid composition but with some common characteristics, as being Arg and Ala among the most abundant amino acids in the wines.

Regarding the concentration of individual amino acids, Albariño showed lower concentrations than other white varieties reported in the literature [5,28]. For instance, the maximum concentrations observed in the current study for Aspacid, Gluacid, Ser, His, Gly, Thr, Arg, Ala, GABA, Tyr, Val, Ile, Leu, Phe, Orn, and Lys were lower than those reported for Greek varieties, Muscats, Chardonnay and Riesling, the one later blended with other varieties (Table 7). In contrast, the concentrations of Asp, Glu, Met, and Try in the studied Albariño wines were within the intervals reported for other white varieties (Table 7). Interestingly, wines from Godello showed similar concentrations to those observed in Moschofilero and Asyrtiko varieties for several amino acids including Aspacid, Ser, Glu, His, Gly, GABA, Met, Ile, Leu, Phe, and Lys (Table 7). Moreover, the maximum concentrations of amino acids found in Godello wines were within the intervals reported for other white varieties, such as Roditis, Muscats, and Riesling (Table 7). The amino acid concentrations observed in Treixadura wines were within the intervals reported for all the varieties displayed in Table 7, being the maximum concentrations of the current study greater than those of Debina, Moschofilero, and Asyrtiko varieties, whereas Treixadura wines presents lower amino acids concentrations than those reported for Chardonnay [5]. The use of several varieties for blending with Riesling increases the variability in the concentrations of amino acids within these wines [28]; as a consequence, the values observed for Albariño, Godello, and Treixadura wines were within the ranges reported for Riesling in the case of most amino acids (Table 7).

Studies referred to the amino acid profiles of Albariño, Godello, and Treixadura wines do not abound [12,13,14], especially for commercial samples. Moreover, most studies that report amino acid concentrations in these varieties refer to grapes or to total contents in wines. In Portugal, a research assessed the influence of nitrogen composition in musts on the contents of volatile sulphur compounds in wines from several grapevine varieties, including Albariño and Treixadura [29]. This research reported total amino acid concentrations in Treixadura musts to be, approximately, three times greater than in Albariño, agreeing with the observations from the current study. Another research performed on sparkling wines from Godello [30], among other varieties, reported amino acid concentrations lower than those observed in the current study for dry wines from the same variety. This disagreement may have been caused by several factors such as the origin and maturation stage of the grapes, the winemaking protocol employed in both studies (including yeast strains used for fermentation), etc. Nevertheless, amino acids such as Pro, GABA, Tyr, Met, Gluacid, and Orn were observed in concentrations within the intervals reported in the current study. Finally, a comparison with results from a previous research on Godello from Valdeorras DO [14], a region located at approximately 120 km inland from Ribeiro, indicated that the amino acid composition of Godello wines was similar, with slightly higher concentrations of Aspacid, GABA, Pro, and Orn in wines from Valdeorras in relation to the intervals reported in the current study.

When compared with the amino acid composition of red wines (Supplementary Table S4), in general, wines from Albariño and Godello tended to have lower concentrations of all individual amino acids while Treixadura tended to have similar concentrations than those reported for red varieties. A more detailed analysis of Supplementary Table S4 indicated that the concentrations of Gluacid, His, Thr, Tyr, Phe, and Lys in Albariño wines were similar as to those found previously in wines from red varieties such as Tempranillo, Monastel and Maturana Tinta [9], while some other amino acids were present in concentrations within the intervals reported for Cabernet Sauvignon [31] (Ala, Val, and Lys) and Touriga Nacional [32] (Gluacid and His) wines. The rest of amino acids in Albariño wines were present at lower concentrations than those reported for red varieties. Regarding Godello wines, Aspacid concentrations tended to be greater than those observed in Tempranillo [9,33], Monastel, and Maturana Tinta [9], while similar to the rest of the red varieties (Supplementary Table S4). Concentrations of Asp, Ser, His, Thr, Ala, Tyr, Val, Met, Ile, Leu, Phe, Orn, and Lys in Godello wines were within the intervals reported for Monastel and Maturana Tinta, whereas the amino acids concentrations reported for wines from other varieties such as Cabernet Sauvignon [31], Touriga Nacional [32], Monastrell [8], and monovarietal and commercial wines from Alentejo [27] tended to be greater than those observed for Godello wines. Finally, Treixadura wines showed concentrations of Aspacid, Leu, and Lys greater than Tempranillo [9,33], Monastel and Maturana Tinta [9] wines; in contrast, His and Arg concentrations were lower than those found in Cabernet Sauvignon [31] and Alentejo [27] wines.

Regarding the total concentration of amino acids, Albariño showed lower values than the white varieties reported in the literature [5,28], as reflected in Table 7. Godello showed similar concentrations as Asyrtiko and Moschofilero varieties (Table 7); whereas Treixadura wines were more similar to Roditis and Debina varieties (Table 7). Étievant et al. [34] reported amino acid concentrations ranging from 126 to 172 mg L^−1^ in red wines from three French regions, higher than the concentrations observed for Albariño and Godello in the current study. Using wines from a French variety, Cabernet Sauvignon, Wang et al. (2014) [31] observed concentrations of amino acids greater than 300 mg L^−1^ when not accounting for Pro, which are twice as high as those found in the current study, likely because they referred to a red variety. Moreover, Ali et al. [28] reported amino acid concentrations in white wines ranging from 112.9 to 3609.5 mg L^−1^ when not accounting for Pro, values much higher than those described in the current work. Furthermore, Arrieta and Prats-Moya [8] detected values from 126 to 484.9 mg L^−1^ in red wines. Nevertheless, Soufleros et al. [5] reported a wide range of amino acid concentrations in Greek white wines (68.4 to 2170 mg L^−1^), in which the concentrations observed in the three varieties considered in the current study would fit. Albariño wines from the current study showed total amino acid concentrations similar to those reported for red wines from Maturana Tinta [9] but lower than those reported for other red varieties [8,9,27,31,32,33]. Godello wines had total amino acid concentrations within the intervals reported for red wines from Monastrell [8], Touriga Nacional [32], and Tempranillo [33], while Treixadura wines had total amino acid concentrations greater than those reported for Maturana Tinta [9] and within the intervals reported for other red varieties including Cabernet Sauvignon [31], Monastel [9], Monastrell [8], Touriga Nacional [32], Tempranillo [9,33], and both monovarietal and commercial wines from the Alentejo region [27]. This proves the large variability that exists in amino acid concentrations in wines, which may originate from many causes including fermentation, grape variety, geographical origin, climatic conditions and viticultural and enological practices used during winemaking, as previously stated by Soufleros et al. [5].

Indeed, the effects of grapevine variety, year and region of production (including agricultural and enological practices) are relevant for the final concentration of amino acids in white wines [5,9,27,28], leading to a large variability in amino acid composition of wines from a given variety. In the current study, we removed the effect of the region, agricultural and enological practices by employing three white varieties grown on the same vineyard, under the same agricultural practices and using the same winemaking procedure. Therefore, the differences in the concentration of amino acids among wines from the different varieties considered in this study will be exclusively originated by the variety and the year.

The concentrations of most amino acids were similar in Albariño and Godello wines, whereas wines from Treixadura were characterized by higher amino acid concentrations, in some cases doubling those of Albariño and Godello. However, when compared to other white varieties, the amino acid concentrations in wines from these three Galician varieties are much lower. For instance, wines produced with Roditis (a Greek variety) showed 430 mg L^−1^ of free amino acids, with Arg and Lys being predominant [5]. The amino acid concentrations in Albariño, Godello, and Treixadura wines are, respectively, 13.8%, 20.7%, and 37.2% that of Roditis wines. Similarly, wines from Chardonnay showed amino acid concentrations between 265 mg L^−1^ [35] and 618 mg L^−1^ [5], significantly higher than those observed in the wines from the varieties studied here. Wines from German varieties such as Riesling, Silvaner or Müller-Thurgau showed higher amino acid concentrations [28] than those observed in Albariño, Godello, and Treixadura wines. Gómez-Alonso et al. [20] reported concentrations from 115 to 570 mg L^−1^ of free amino acids in wines from the Airén variety, in which Gluacid, Arg, Ala, GABA and Lys predominated. Treixadura wines, with 159.83 mg L^−1^, fit within this range, whereas Albariño and Godello showed lower concentrations of free amino acids, 59.50 and 88.99 mg L^−1^, respectively. These results are particularly relevant because the total concentration of amino acids has a direct relation with the synthesis of aroma compounds (such as esters and acetates) during fermentation [36,37]; however, this depends on the grapevine variety [37]. Therefore, some of the differences in aroma composition reported for the three varieties considered in the current study [10,11] might be explained by the variation in their amino acid profiles.

In the current work, we isolated the effect of variety and year by using experimental wines coming from the same vineyard, which was equally managed for the three varieties considered. However, the limited sample size used in the current study (3 wines per variety and year) prevents from obtaining general conclusions about the amino acid profile of Albariño, Godello and Treixadura wines from commercial wineries, especially those from other regions, due to the large number of factors involved in amino acid profiles [5,6]. Nevertheless, the statistical methods employed have sufficient power for discriminating among the samples studied and, consequently, allowing for a differentiation by variety. Type I error was fixed to 5%, whereas type II error (the power of statistical tests [38]) was dependent on the sample size, magnitude of effects and the precision of the determinations performed. The precision of the analytical method employed was high [6,20] and we analyzed the samples in triplicate, thus improving accuracy. This fact, along with the low magnitude in the differences among varieties for the concentrations of a given amino acid increased the power of the statistical tests used. Furthermore, the sample size employed limited the use of other statistical techniques such as partial least regression [39], which are common in chemometrics [40]; therefore, we employed the most appropriate statistical methods for analyzing our data.

The 22 analyzed amino acids appeared in the wines from the three Galician varieties and in the three studied years, with slight differences in the relative proportion of major amino acids, except for Aspacid, Gluacid and Lys. Instead, the profiles and contents of the minor amino acids differed clearly according to the variety, which could serve as potential tool for classification of wines. When principal component analysis, using the percentages of 21 amino acids as variables (excluding Pro), was applied, a marked trend to group wines in relation to varietal characteristics was achieved. The first two principal components explained 52% of the total variance. In the bi-plot, Albariño samples were positioned in the lower part of the graph, while those of Treixadura were in the upper part. Godello samples appeared in the middle of the plot, between those from the other two varieties. This clearly indicates that the amino acid profiles can be useful tools for discerning the origin of wines, confirming previous studies [5,6,9].

## 5. Conclusions

In this work, for the first time, the amino acid profiles of Albariño, Godello, and Treixadura wines were analyzed. Although the studied grapevine varieties presented similar qualitative compositions (the major and minor amino acids were common), Treixadura wines were characterized by the highest concentrations. Most differences among these three varieties were observed in minor amino acids. Principal component analysis was able to establish a clear differentiation between these three Galician varieties. Moreover, the amino acid concentrations of these white wines were lower than the values reported for other white wines. Therefore, this study confirms that some factors, such as grape variety, geographic location or vintage affect the amino acid composition of grapes and, consequently, wines and the amino acid profiles could be used for wine discrimination.

## Figures and Tables

**Table 1:** Mean air temperature and total rainfall at the studied vineyard during the maturation period (August and September until harvest) in 2012, 2013, and 2014.

Year	Mean Temperature (°C)		Total Rainfall (mm)	
	August	September	August	September Till Harvest
2012	19.8	19.0	22.8	0.2
2013	21.8	19.6	0.0	2.6
2014	19.9	18.9	29.0	12.4

**Table 2:** Yield components and pruning weight for the three white grapevine varieties studied. Data are averages for the three years ± standard errors. The *p*-values for the variety and year factors and their interaction are shown.

Variety	Cluster Number	Yield	Cluster Weight	Pruning Weight
		kg vine^−1^	g	kg vine^−1^
Albariño	41.4 ± 1.9 b	3.1 ± 0.2 a	73.6 ± 2.8 a	1.5 ± 0.1 b
Godello	41.9 ± 1.9 b	5.4 ± 0.3 b	133.9 ± 3.7 b	1.5 ± 0.1 b
Treixadura	21.6 ± 1.1 a	3.6 ± 0.3 a	155.2 ± 6.1 c	0.9 ± 0.1 a
Factors				
Variety	<0.01	<0.01	<0.01	<0.01
Year	0.022	<0.01	<0.01	0.026
Variety × Year	0.051	0.024	0.918	0.651

Different letters in the column indicate significant differences among varieties for a given parameter according to the Tukey’s HSD test.

**Table 3:** General parameters of the musts from the three white grapevine varieties studied. Data are averages for the three years ± standard errors. The *p*-values for the factors variety, year, and their interaction are shown.

Variety	Total Soluble Solids	pH	Total Acidity	Tartaric Acid	Malic Acid
	◦ Brix		g L^−1^ as Tartaric Acid	g L^−1^	g L^−1^
Albariño	22.9 ± 0.1	3.08 ± 0.02 a	8.1 ± 0.1 c	8.3 ± 0.3 b	3.0 ± 0.2 b
Godello	23.5 ± 0.3	3.27 ± 0.03 b	6.8 ± 0.3 b	8.1 ± 0.3 b	2.4 ± 0.1 a
Treixadura	23.3 ± 0.5	3.54 ± 0.04 c	5.5 ± 0.2 a	6.1 ± 0.2 a	2.9 ± 0.2 b
Factors					
Variety	0.464	<0.01	<0.01	<0.01	<0.01
Year	0.223	0.090	0.099	0.180	0.032
Variety × Year	0.370	0.520	0.054	0.025	0.324

Different letters in the column indicate significant differences among varieties for a given parameter according to the Tukey’s HSD test.

**Table 4:** General parameters of the wines from the three white grapevine varieties studied. Data are averages for the three years ± standard errors. The *p*-values for the factors variety, year and their interaction are shown.

Variety	Alcohol	pH	Total Acidity	Tartaric Acid	Malic Acid
	% Vol.		g L^−1^ as Tartaric Acid	g L^−1^	g L^−1^
Albariño	13.6 ± 2.2	3.03 ± 0.30 a	9.1 ± 1.8 b	4.8 ± 1.9 b	2.7 ± 1.3 b
Godello	14.1 ± 0.2	3.15 ± 0.04 a	7.1 ± 0.2 a	3.0 ± 0.4 a	2.0 ± 0.1 a
Treixadura	13.9 ± 0.3	3.45 ± 0.05 b	6.9 ± 0.2 a	2.5 ± 0.3 a	2.7 ± 0.1 b
Factors					
Variety	0.300	<0.01	<0.01	<0.01	<0.01
Year	0.105	0.047	0.769	0.088	< 0.01
Variety × Year	0.466	0.773	0.079	0.094	0.356

Different letters in the column indicate significant differences among varieties for a given parameter according to the Tukey’s HSD test.

**Table 5:** Concentrations of free amino acids in the wines from the three white grapevine varieties studied. Data are averages for the three years ± standard errors. The *p*-values for the factors variety, year and their interaction are shown.

Compound	Albariño	Godello	Treixadura	Factors		
	mg L^−1^			Variety	Year	Variety × Year
Aspacid	7.76 ± 0.31 a	9.86 ± 0.93 a	17.25 ± 2.30 b	<0.01	0.505	0.654
Gluacid	6.63 ± 0.83 a	12.20 ± 3.32 b	14.42 ± 3.55 b	<0.01	<0.01	0.144
Asp	4.13 ± 0.88 a	5.73 ± 1.00 a	9.54 ± 1.08 b	<0.01	<0.01	0.721
Ser	2.10 ± 0.18 a	3.58 ± 0.53 b	6.04 ± 0.88 c	<0.01	0.025	0.516
Glu	0.28 ± 0.04 a	0.35 ± 0.05 a	1.08 ± 0.17 b	<0.01	0.558	0.801
His	3.61 ± 0.21 a	4.41 ± 0.48 a	7.04 ± 0.83 b	<0.01	0.055	0.534
Gly	2.83 ± 0.28 a	3.70 ± 0.28 a	6.02 ± 0.70 b	<0.01	0.429	0.477
Thr	2.40 ± 0.16 a	2.42 ± 0.26 a	4.11 ± 0.70 b	0.025	0.858	0.697
Arg	4.26 ± 0.30 a	5.92 ± 0.79 a	10.41 ± 1.38 b	<0.01	0.045	0.081
Ala	3.96 ± 0.45 a	6.38 ± 0.97 b	11.97 ± 1.76 c	<0.01	0.199	0.264
GABA	3.53 ± 0.34 a	3.89 ± 0.74 a	11.13 ± 0.82 b	<0.01	<0.01	0.064
Pro	175.9 ± 69.9 a	298.3 ± 93.8 a	1652.6 ± 264.7 b	<0.01	0.766	0.285
Tyr	1.02 ± 0.25 a	1.69 ± 0.34 a	3.08 ± 0.92 b	<0.01	<0.01	0.037
Val	2.66 ± 0.25 a	5.14 ± 0.96 a	10.49 ± 1.94 b	<0.01	0.278	0.486
Met	1.02 ± 0.13 a	1.45 ± 0.29 ab	2.63 ± 0.56 b	0.012	0.053	0.529
Cys	0.00 ± 0.00 a	0.00 ± 0.00 a	0.13 ± 0.08 a	0.111	0.413	0.509
Ile	1.15 ± 0.10 a	1.96 ± 0.30 a	3.93 ± 0.82 b	<0.01	0.364	0.543
Try	0.68 ± 0.14 a	0.66 ± 0.05 a	1.14 ± 0.16 b	<0.01	<0.01	0.077
Leu	3.62 ± 0.36 a	6.26 ± 0.93 b	11.71 ± 1.93 c	<0.01	0.582	0.668
Phe	2.63 ± 0.25 a	4.26 ± 0.57 b	7.44 ± 1.10 c	<0.01	0.729	0.626
Orn	0.37 ± 0.07 a	0.56 ± 0.10 a	3.06 ± 2.57 b	<0.01	0.209	0.202
Lys	4.85 ± 0.52 a	8.57 ± 1.40 b	17.22 ± 2.79 c	<0.01	0.316	0.636
Total	235.4 ± 72.2 a	387.3 ± 104.5 a	1812.5 ± 283.1 b	<0.01	0.383	0.594
Total-Pro	59.50 ± 3.95 a	88.99 ± 12.90 b	159.83 ± 20.78 c	<0.01	0.383	0.594

Different letters in the row indicate significant differences among varieties for a given amino acid according to the Tukey’s HSD test. Abbreviations: Alanine (Ala), Asparagine (Asp), Aspartic acid (Aspacid), Arginine (Arg), Cysteine (Cys), γ-aminobutyric acid (GABA), Glutamic acid (Gluacid), Glutamine (Glu), Glycine (Gly), Histidine (His), Isoleucine (Ile), Leucine (Leu), Lysine (Lys), Methionine (Met), Ornithine (Orn), Phenylalanine (Phe), Proline (Pro), Serine (Ser), Threonine (Thr), Tryptophan (Try), Tyrosine (Tyr), Valine (Val). Total-Pro (Total amino acid concentration except proline).

**Table 6:** Minimum and maximum concentrations (mg L^−1^) of free amino acids in the wines from the three white grapevine varieties over the study period (2012–2014).

Compound	Albariño		Godello		Treixadura	
	Minimum	Maximum	Minimum	Maximum	Minimum	Maximum
Aspacid	5.84	8.71	5.55	13.95	10.95	28.87
Gluacid	3.02	9.94	1.01	27.38	5.67	35.19
Asp	0.68	8.85	2.75	10.96	3.36	13.12
Ser	1.21	3.09	2.24	6.59	3.62	10.51
Glu	0.13	0.48	0.21	0.64	0.42	2.05
His	2.75	4.40	2.46	6.76	3.65	11.66
Gly	1.44	3.79	2.49	5.14	3.38	10.41
Thr	1.70	3.01	1.52	3.78	2.36	8.43
Arg	2.94	5.85	3.10	9.70	4.37	18.64
Ala	2.25	5.81	2.56	10.22	7.00	24.40
GABA	1.46	4.91	0.89	7.18	6.92	14.56
Pro	1.20	503.72	2.65	743.02	703.68	3016.23
Tyr	0.18	2.00	0.34	3.03	0.69	9.43
Val	1.74	3.84	2.19	10.47	4.81	21.46
Met	0.35	1.62	0.42	3.01	0.99	5.32
Cys	ND	ND	ND	ND	ND	0.71
Ile	0.60	1.48	1.17	3.86	1.62	8.13
Try	0.27	1.35	0.52	0.97	0.67	2.04
Leu	1.50	4.97	2.45	10.82	6.04	22.63
Phe	1.50	3.89	1.70	6.93	4.22	13.78
Orn	0.13	0.68	0.15	0.92	0.31	23.60
Lys	2.02	7.12	3.45	16.15	8.64	31.22
Total	38.70	576.75	45.20	898.27	799.67	3266.82
Total-Pro	37.50	73.03	41.65	152.25	95.99	269.78

Abbreviations: Alanine (Ala), Asparagine (Asp), Aspartic acid (Aspacid), Arginine (Arg), Cysteine (Cys), γ-aminobutyric acid (GABA), Glutamic acid (Gluacid), Glutamine (Glu), Glycine (Gly), Histidine (His), Isoleucine (Ile), Leucine (Leu), Lysine (Lys), Methionine (Met), Ornithine (Orn), Phenylalanine (Phe), Proline (Pro), Serine (Ser), Threonine (Thr), Tryptophan (Try), Tyrosine (Tyr), Valine (Val). Total-Pro (Total amino acid concentration except proline). ND (Not detected).

**Table 7:** Concentrations (mg L^−1^) of amino acids reported in the literature for wines from several white varieties. When available in the literature, minimum–maximum intervals are reported, otherwise, values are averages ± standard deviations, as reported in the references cited. Abbreviations are the same as for Table 6.

Compound	Roditis	Debina	Moschofilero	Asyrtiko	Muscat d’Alexandrie	Muscat White	Chardonnay	Riesling Blended
	[5]	[5]	[5]	[5]	[5]	[5]	[5]	[28]
Aspacid	26.7 ± 9.7	14.9 ± 9.0	10.1 ± 4.5	9.1 ± 6.1	23.8 ± 8.3	14.6 ± 3.5	36.9 ± 26.3	ND
Gluacid	35.5 ± 13.8	25.5 ± 9.7	14.0 ± 6.4	16.0 ± 7.9	33.2 ± 10.3	18.9 ± 7.3	58.2 ± 55.3	ND
Asp	10.7 ± 5.4	7.0 ± 6.2	7.1 ± 3.0	5.5 ± 4.8	5.8 ± 3.5	5.5 ± 4.4	18.1 ± 18.5	12.0–239.0
Ser	10.4 ± 4.0	6.2 ± 4.4	5.3 ± 1.1	4.1 ± 1.7	13.2 ± 11.9	12.0 ± 8.9	18.6 ± 19.4	2.4–140.0
Glu	0.8 ± 1.1	0.8 ± 1.5	0.8 ± 1.1	0.5 ± 0.9	0.7 ± 1.1	0.8 ± 1.3	0.7 ± 0.9	25.2–419.0
His	14.4 ± 5.7	7.7 ± 5.8	5.4 ± 1.9	5.5 ± 3.5	20.4 ± 24.3	16.0 ± 9.5	18.5 ± 16.2	ND
Gly	10.6 ± 3.6	5.5 ± 2.8	6.0 ± 0.7	5.8 ± 1.8	12.9 ± 10.8	14.3 ± 4.9	13.8 ± 6.5	9.3–129.0
Thr	18.5 ± 4.3	11.9 ± 5.1	10.9 ± 4.9	12.3 ± 3.7	21.4 ± 16.8	26.3 ± 9.8	28.3 ± 19.6	2.2–199.0
Arg	75.1 ± 101.0	12.5 ± 9.7	10.9 ± 5.1	11.0 ± 6.4	158.1 ± 371.0	199.0 ± 204.0	132.0 ± 202.0	ND
Ala	30.9 ± 9.5	15.7 ± 11.0	16.3 ± 4.9	16.3 ± 7.3	30.2 ± 15.8	32.6 ± 22.5	83.6 ± 103.0	11.7–337.0
GABA	28.6 ± 34.0	5.2 ± 0.9	6.5 ± 2.3	5.5 ± 2.9	59.6 ± 156.0	125.0 ± 110.0	49.6 ± 71.4	2.5–170.1
Pro	ND	ND	ND	ND	ND	ND	ND	489–1595
Tyr	15.6 ± 7.7	12.1 ± 10.4	5.4 ± 3.9	5.4 ± 3.6	17.8 ± 6.2	10.7 ± 6.9	17.4 ± 13.6	7.1–139.0
Val	12.6 ± 8.2	6.5 ± 5.6	3.7 ± 2.1	2.0 ± 2.0	7.8 ± 2.8	6.5 ± 4.6	12.9 ± 9.4	2.4–54.7
Met	5.1 ± 3.3	3.8 ± 2.5	2.4 ± 0.6	1.9 ± 1.0	4.1 ± 1.5	2.1 ± 1.3	3.7 ± 2.0	2.0–26.3
Ile	8.2 ± 3.5	5.3 ± 4.6	3.5 ± 2.4	2.4 ± 2.3	7.7 ± 1.9	6.4 ± 2.3	6.2 ± 2.4	1.1–39.8
Try	2.2 ± 1.7	2.5 ± 2.3	2.1 ± 1.5	0.7 ± 0.9	3.2 ± 3.0	1.9 ± 3.8	2.3 ± 1.9	ND
Leu	27.7 ± 8.8	15.5 ± 10.2	11.7 ± 5.9	9.1 ± 5.6	23.6 ± 6.8	14.9 ± 4.9	24.0 ± 11.7	1.0–20.1
Phe	20.0 ± 7.2	11.6 ± 7.7	8.3 ± 4.2	7.2 ± 4.4	23.7 ± 13.2	15.0 ± 6.8	17.2 ± 9.0	6.2–129.0
Orn	17.2 ± 17.6	10.8 ± 8.0	4.8 ± 2.0	2.6 ± 1.5	3.9 ± 3.8	11.6 ± 5.0	13.5 ± 16.5	2.4–66.6
Lys	42.8 ± 17.8	23.0 ± 12.8	17.5 ± 8.2	15.7 ± 8.2	39.2 ± 15.0	24.6 ± 11.3	37.1 ± 16.4	4.0–117.0
Total-Pro	430	212	164	151	535	597	619	ND

## Supplementary Materials

The following are available online at https://www.mdpi.com/2304-8158/9/2/114/s1, Table S1: Concentrations of free amino acids (mg L^−1^) in the wines from Albariño for the three years studied (2012–2014). Data are values for each single replication. The total concentration of amino acids as well as the total without proline are also shown., Table S2: Concentrations of free amino acids (mg L^−1^) in the wines from Godello for the three years studied (2012–2014). Data are values for each single replication. The total concentration of amino acids as well as the total without proline are also shown., Table S3: Concentrations of free amino acids (mg L^−1^) in the wines from Treixadura for the three years studied (2012–2014). Data are values for each single replication. The total concentration of amino acids as well as the total without proline are also shown., Table S4: Concentrations (mg L^−1^) of amino acids reported in the literature for wines of red varieties. When available, minimum − maximum intervals are reported, otherwise, values are averages ± standard deviations. Abbreviations are the same as for the former tables (ND = No data). References are cited in the main text.
